# Cloud accelerated alignment and assembly of full-length single-cell RNA-seq data using Falco

**DOI:** 10.1186/s12864-019-6341-6

**Published:** 2019-12-30

**Authors:** Andrian Yang, Abhinav Kishore, Benjamin Phipps, Joshua W. K. Ho

**Affiliations:** 1 Victor Chang Cardiac Research Institute, 405 Liverpool St, Darlinghurst, 2010 New South Wales Australia; 2 St. Vincent’s Clinical School, University of New South Wales, Darlinghurst, 2010 New South Wales Australia; 3 School of Biomedical Sciences, Li Ka Shing Faculty of Medicine, The University of Hong Kong, Pokfulam, Hong Kong China

**Keywords:** Single-cell RNA-seq, Cloud computing, Falco, Alignment, Transcript assembly

## Abstract

**Background:**

Read alignment and transcript assembly are the core of RNA-seq analysis for transcript isoform discovery. Nonetheless, current tools are not designed to be scalable for analysis of full-length bulk or single cell RNA-seq (scRNA-seq) data. The previous version of our cloud-based tool Falco only focuses on RNA-seq read counting, but does not allow for more flexible steps such as alignment and read assembly.

**Results:**

The Falco framework can harness the parallel and distributed computing environment in modern cloud platforms to accelerate read alignment and transcript assembly of full-length bulk RNA-seq and scRNA-seq data. There are two new modes in Falco: alignment-only and transcript assembly. In the alignment-only mode, Falco can speed up the alignment process by 2.5–16.4x based on two public scRNA-seq datasets when compared to alignment on a highly optimised standalone computer. Furthermore, it also provides a 10x average speed-up compared to alignment using published cloud-enabled tool for read alignment, Rail-RNA. In the transcript assembly mode, Falco can speed up the transcript assembly process by 1.7–16.5x compared to performing transcript assembly on a highly optimised computer.

**Conclusion:**

Falco is a significantly updated open source big data processing framework that enables scalable and accelerated alignment and assembly of full-length scRNA-seq data on the cloud. The source code can be found at https://github.com/VCCRI/Falco.

## Background

The main step in most RNA sequencing (RNA-seq) analyses is the alignment of sequencing reads against the reference genome or transcriptome to find the location from which the reads originate. The positional information of the reads, together with the sequences of the reads themselves, forms the basis from which many different downstream analyses can be performed, such as gene expression analysis, variant calling, and novel isoform identification. The read alignment step is typically one of the most time consuming steps during RNA-seq analysis due to the complex algorithm utilised during the read alignment process. There have been a number of recently published tools which are designed to skip this expensive step through the use of pseudoalignment methods, such as kallisto [1] and Salmon [2]. However, these tools are designed specifically for read quantification and therefore are not applicable to other types of downstream analyses.

There are a number of tools which have been published for alignment of RNA-seq reads, including STAR [3], HISAT2 [4] and Subread [5]. While these tools offer parallelisation to perform read alignment in a time-efficient manner, they are typically limited to a single machine only. With the rapidly increasing number of profiles which can be generated by single-cell RNA-seq (scRNA-seq) techniques, there is a need to develop tools which can perform read alignment of large datasets across many machines in a scalable manner. We have previously developed the Falco framework for scalable analysis of scRNA-seq data on the cloud [6], with the initial version of Falco being primarily designed for the quantification of scRNA-seq datasets. While most downstream analysis of scRNA-seq datasets are based on gene expression, there are other types of downstream analyses which does not require gene expression, including novel isoform identification and immune cell receptor reconstruction. In order to enable the Falco framework to support these types of downstream analyses, we introduce an alignment-only mode which produces alignment information output for individual scRNA-seq samples.

The idea of parallelising read alignment across distributed computing infrastructure is not novel – there are already existing tools available that perform read alignment on cluster computing, grid computing and cloud computing infrastructures. Within the context of tools developed using Big Data frameworks, there are Hadoop-based tools, such as Halvade-RNA [7] and HSRA [8], and Spark-based Rail-RNA [9], for alignment of spliced reads. Halvade-RNA is mainly designed for variant calling of RNA-seq data using STAR aligner and GATK [10] variant caller, though it can optionally produce alignment information output. HSRA, on the other hand, is designed for RNA-seq alignment using the HISAT2 aligner. These two tools will not be able to properly analyse the large number of samples present in scRNA-seq data as they are mainly designed to process individual samples. In contrast, Rail-RNA is able to perform multi-sample alignment of RNA-seq data using a modified Bowtie algorithm [11] to handle spliced reads. One limitation of Rail-RNA is that the alignment tool used is non-configurable, unlike the Falco framework, which allows the user to customise the alignment tool used. Furthermore, Rail-RNA requires the user to manually pre-process the sequencing reads by themselves, whereas the Falco framework provides a pre-processing step as part of the analysis.

The downstream analyses following the read alignment steps typically make use of transcript information to provide biological context for the aligned reads. For example, in feature quantification, transcript information is used as feature to summarise reads into counts representing transcript abundance. For eukaryotic genome, the transcript information provides multiple levels of granularity as genes can go through alternative splicing, whereby multiple isoforms of proteins are generated from the same precursor mRNA through exclusion or inclusion of exonic regions. Alternative splicing is a commonly occurring process within the human genome, with >95% of the multi-exonic genes having 2 or more isoforms [12], and the different isoforms of proteins typically have unique functionality. Some isoforms are expressed only in specific cell types [13] and novel isoforms arising from mutations may result in diseases such as cancer [14].

Current methods of isoform analysis are largely dependent on existing transcript isoform information from reference annotation, such as those published by ENCODE and UCSC. However, there are limitations with using reference annotation as we are restricted to studying known transcripts only. While this is less of an issue in human and well-annotated model organisms, isoform analysis will not be as accurate for non-model organisms or organism with limited/partial annotation information. Moreover, novel isoform which may arise due to mutation will not be detectable when using existing annotation. In order to alleviate the problem of detecting new isoforms for isoform analysis, transcript assembly can be utilised to detect and update existing annotations with novel isoforms.

As the name implies, transcript assembly is the process of recovering transcript sequences through assembly of reads. There are two types of approaches for performing transcript assembly - genome-guided transcriptome assembly and de novo transcriptome assembly. In genome-guided transcriptome assembly, read alignment information is used to create read overlap graphs for computing transcripts isoforms. By comparison, the de novo transcriptome assembly approach uses the sequence of the reads to construct De Bruijn graphs for computation of transcripts isoforms. The genome-guided approach is more suited to studying gene isoforms in organism with high quality reference genomes, while the de novo approach is more suitable when the reference genome is not available or is of poor quality, and for studying isoforms of genes with high degree of editing and/or splicing, such as in immune genes. Cufflinks [15], StringTie [16] and Scallop [17] are examples of tools utilising genome-guided approach. Tools which utilises de novo transcriptome assembly approach include Trinity [18], Trans-ABySS [19] and Oases [20].

Current tools for transcriptome assembly are mainly designed for bulk RNA-seq datasets and will not scale for analysing scRNA-seq datasets. There are a small number of tools which are designed specifically for scRNA-seq such as BASIC [21] and V(D)J Puzzle [22], though they are limited to reconstructing immune cell (B- and T-cell) receptors for study of immune-repertoire diversity. Furthermore, some of these tools have limited paralellism, with BASICS supporting only parallelisation on a single machine. V(D)J Puzzle, on the other hand, supports parallelisation on a single machine and on a cluster computing environment. Given the lack of a scalable transcriptome assembly tools for scRNA-seq which can support full transcriptome assembly, we have also introduced a transcriptome assembly analysis feature into the Falco framework to enable the assembly of full transcriptomes for large datasets in a scalable manner. Another benefit of including transcript assembly analysis is the creation of a more accurate gene annotation which can then be used by the Falco framework for more accurately quantifying gene and/or isoform expression.

In this paper, we describe the development of the Falco framework which incorporates two additional modes of analysis: (1) alignment-only mode, where the output is an alignment file for each sample, and (2) transcript assembly mode, where the output is a reconstructed transcript isoform annotation based on the data. Collectively, these new modes will enable Falco to be a comprehensive, scalable bioinformatics platform for processing full-length single-cell RNA-seq data.

## Implementation

**Fig. 1:**
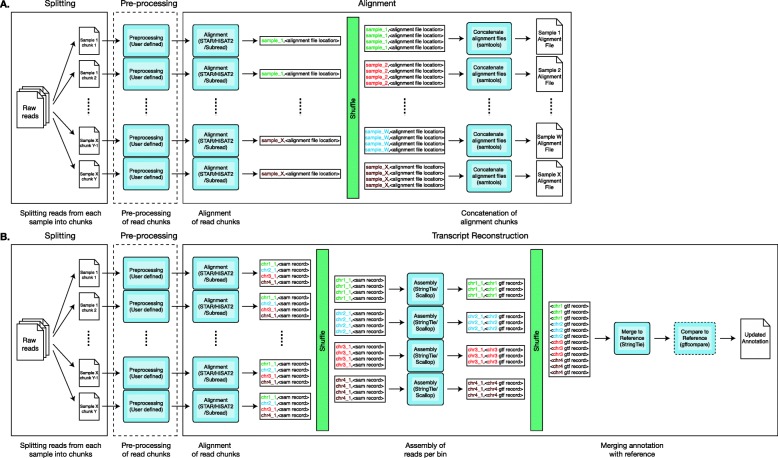
Overview of the Falco framework pipelines. **a** Alignment-only pipeline. The pipeline is composed of the splitting and pre-processing steps from the original Falco framework and the new Spark-based alignment step from the Falco framework. The alignment step is composed of two stages - an alignment stage, where read chunks are aligned and stored in a temporary location in HDFS, and a concatenation stage, where alignment chunks from the same sample are concatenated to obtain the full alignment result. **b** Transcript-assembly pipeline. The pipeline is also composed of the splitting and pre-processing steps from the original Falco framework in addition to the new Spark-based transcript assembly step from the Falco framework. The transcript assembly step is composed of a number of stages, including an alignment stage, which performs alignment of read chunks and binning of the alignment result; an assembly stage which perform transcript assembly in parallel, and a merging step, where assembled transcripts are merged with the reference annotation to produce an updated annotation

The initial version of the Falco framework is composed of three steps - a splitting step for splitting and interleaving of input fastq files into read chunks, an optional pre-processing step for performing pre-processing of the read chunks and an analysis step for alignment and quantification of the read chunks. To implement the alignment-only mode within the Falco framework, we have a designed a new alignment analysis step to replace the read quantification analysis step in the Falco framework (Fig. 1a). The alignment analysis step takes in the same read chunks input as the previous read quantification analysis step and will output a single alignment file for each sample into either S3 or HDFS, depending on the output location specified by user. Similarly, the transcript assembly was implemented through the creation of a new transcript assembly step which performs alignment of sequencing reads followed by assembly of transcripts (Fig. 1b). The genome-guided transcript assembly approach was chosen over the de novo transcript assembly approach due to the high computational cost of de novo assembly and the complexity of adapting existing de novo transcript assembly tools to work with the parallelisation approach utilised by Falco. The input of the transcript assembly step is the read chunks input used by both the read quantification and alignment analysis steps, with the output of the step being an annotation file containing the assembled transcript.

As with the read quantification analysis step, both the alignment analysis step and the transcript assembly step are configurable by the user. The alignment analysis step currently supports both STAR or HISAT2 as the aligner, with the transcript assembly step also supporting STAR or HISAT2 as the aligner and either StringTie or Scallop as the transcript assembly tool. Users can also further customise the Falco pipeline by adding custom alignment and/or transcript assembly tools, similar to the customisation options provided by the initial version of the Falco framework. New submission scripts have also been created to allow users to easily submit the two analysis steps to the EMR cluster.

### Alignment-only mode

The alignment analysis step is a Spark job which consist of two stages - alignment of read chunks, followed by concatenation of the aligned chunks. In the alignment stage, the interleaved reads within the read chunks are first converted to FASTQ file format so that it can be read by the alignment tool. The alignment tool - STAR or HISAT2 - is then executed using Python’s built-in subprocess library in order to perform alignment of reads against the reference genome. The output of the alignment tool is a BAM alignment file in the case of STAR and a SAM alignment file in the case of HISAT2. As such, an extra processing step of converting SAM to BAM using Samtools is required when HISAT2 is used as the alignment tool. The binary-based BAM file format is chosen over the text-based SAM file format due to the space efficiency of the BAM format, which is achieved through compression of alignment records. The alignment chunk is then uploaded to a temporary location within HDFS or S3 and the location of the alignment chunk is output, together with the sample name from which the read chunks originate.

A shuffling process is then performed to group together the locations of the alignment chunks per sample. This is followed by a concatenation stage that combines the alignment chunks into a single alignment file for each sample. During the concatenation stage, the alignment chunks are iteratively copied from the temporary location into the local disk and concatenated to a previously concatenated file using Samtools. The iterative concatenation of alignment chunks is chosen over batch concatenation of the alignment chunks due to the constraint of disk space available in the worker since there can be an arbitrary amount of chunks for a single sample. Once all the chunks are concatenated into a single alignment file, it is then uploaded to the output location specified by the user, which can either be in S3 or HDFS. Finally, the alignment chunks stored in the temporary location are deleted to free up the space for the next analysis

### Transcript assembly mode

The transcript assembly step is implemented as a Spark job consisting of four stages - alignment of read chunks, assembly of reads per bin, merging of assembled transcripts against the reference annotation and, optionally, comparison of the updated annotation against the reference annotation. The first stage – alignment of read chunks – is implemented in a similar manner to the alignment stage in the alignment analysis step, where read chunks are aligned against the reference genome using either STAR or HISAT2. However, unlike the alignment analysis step, the aligned reads are not stored in a temporary location, but rather each alignment record is output together with the names of the bins that overlap that particular read. The bin names are calculated based on the locations where the reads align to in the genome and each read may be output multiple times depending on the number of bins that it overlaps. In order to reduce the amount of data that needs to be shuffled, the read sequence and the sequence quality was removed from the alignment record as this information is not utilised in the transcript assembly process.

The alignment records are then shuffled in order to group records from the same bins together. This is followed by an assembly stage where the alignment records are written to an alignment file and sorted by co-ordinate using Samtools [23]. The transcript assembly tool – StringTie or Scallop – is then executed using Python’s subprocess library to perform genome-guided transcript assembly with the sorted alignment file as input. Depending on the transcript assembly tool chosen, users can also choose to utilise the reference annotation when performing transcript assembly. In this case, a partial annotation file, created by filtering the reference annotation to select only transcripts located in the chromosome of the bin being processed, is included as an input when executing StringTie. The annotation filtering step is performed to reduce both the execution time and the amount of output produced by StringTie, as it only needs to consider a smaller subset of reference transcripts during transcript assembly. After execution of the transcript assembly tool, the assembled transcripts are then output together with the name of the bin.

The transcripts then undergo another shuffling process in order to sort the transcripts by the bin names and to group the transcripts across all bins. The aggregated transcripts are collected into the main ’driver’ executor where it is passed into the merging stage. In the merging stage, the transcripts are first written into an annotation file, followed by execution of StringTie in GTF merge mode using both the assembled annotation file and reference annotation file as input. The resulting merged (updated) annotation file, containing both the reference transcripts and newly assembled transcripts, is then uploaded to the location specified by user in either S3 or HDFS.

The transcript assembly step also has an optional fourth stage that performs comparison of the merged annotation against the reference annotation using the GffCompare tool [16]. GffCompare will calculate the sensitivity and precision metrics of the updated annotation as compared to the reference annotation at base, exon, intron, intron chain, loci and transcript levels. The comparison statistics produced by the comparison tool will also be uploaded to the location specified by the user.

## Results

### Evaluation of Falco alignment-only mode

One of the features of the read-quantification mode in the initial version of the Falco framework is the production of the gene expression matrix that is identical to that produced in a sequential analysis, where reads are not split into smaller chunks. This was achieved through careful selection of tools that are known to be deterministic (STAR, HTSeq [24] and featureCounts [25]) or by adjusting the parameters of the tool to ensure the output produced is deterministic (HISAT2). As such, it will be ideal for the alignment-only mode to also produce alignment outputs that are identical to those produced in a sequential analysis. In order to test this hypothesis, 100 files were randomly selected from both the mouse embryonic stem cell (ESC) single cell dataset and the human brain single cell dataset, and then aligned using either sequential alignment on a single node and Falco. The alignment file produced by the two different approaches were then compared to see if the outputs produced are identical. The comparison was performed by first sorting the alignment files by their read name using Samtools, followed by running the diff command with the two alignment files as input.

The result of the comparison shows that the alignment files produced with STAR as the alignment tool contain identical alignment records when run through either Falco or sequentially, with some minor difference in the header of the alignment file due to the inclusion of the command used for running STAR in the program (PG) and text command (CO) records. In contrast, the alignment records produced by HISAT2 with default parameters shows some differences between Falco-based and sequential runs due to HISAT2 being non-deterministic. Therefore, the –tmo parameter was again used when running HISAT2 in order to make HISAT2 produce deterministic output by performing alignment within known transcripts only. The result of the comparison when running HISAT2 with the –tmo parameter shows that the alignment files produced contains identical alignment records, with a minor difference in the value of the PG record in the header of the alignment.

### Scalability of Falco alignment-only mode

In order to evaluate the performance of the Falco alignment-only analysis, a runtime comparison was performed for STAR and HISAT2 using two single-cell RNA-seq datasets with and without using the Falco framework, similar to the evaluation done for the initial version of the Falco framework. As with the evaluation of the Falco framework, the single-cell RNA-seq datasets used are a mouse embryonic stem cell (ESC) single cell dataset, containing 869 samples of 200 bp paired-end reads, stored in 1.02 Tb of gzipped FASTQ files [26]; and a human brain single cell data containing 466 samples of 100 bp paired-end reads stored in 213.66 Gb of gzipped FASTQ files [27]. We utilised the same configuration for analysis in a single computing node - ranging from the naive single processing approach to a highly parallelised approach - and for the size of the EMR clusters - ranging from 10 to 40 nodes, together with the same AWS EC2 instance type for single node (r3.8xlarge) and Falco cluster (master - r3.4xlarge, core - r3.8xlarge). For a fair comparison between the single-node based runs and the Falco runs, the timing for alignment on the Falco framework includes the timing for both the cluster set-up and FASTQ splitting step as these pre-processing steps are only necessary when performing alignment using the Falco framework.

Performing alignment using STAR on a single node with differing parallelisation approaches results in runtimes ranging from 35 h down to 20 h for the mouse dataset and 11 h to 5 h for the human dataset. In contrast, the runtime for alignment using STAR on the Falco framework ranges from 8 h down to just 3.5 h for the mouse dataset and 1.7 h down to less than an hour for the human dataset, representing a minimum speed up of 2.5x (10 nodes vs 12 processes for the mouse dataset) up to 15.8x (40 nodes vs 1 process for the human dataset) (Table 1). Similarly, performing alignment using HISAT2 on a single node with differing parallelisation approach results in a minimum runtime of 15 h and 3 h for the mouse and human datasets, respectively, with the mouse dataset taking close to 2 days to run on 1 process. Falco, on the other hand, was able to complete the alignment for the mouse dataset in less than 6 h and the human dataset in less than 1.2 h, representing a speed up ranging from 2.5x (10 nodes vs 16 processes for the human dataset) up to 16.4x (40 nodes vs 1 processes for the mouse dataset) (Table 1).

**Table 1:** Runtime comparison for alignment of single cell datasets with and without the Falco framework

System	Nodes	Mouse - embryonic stem cell (hours)		Human - brain (hours)	
		STAR	HISAT2	STAR	HISAT2
Standalone	1 (1 process)	34.9	42.7	11.1	9.8
	1 (12 processes)	20.2	14.9	5.2	3.1
	1 (16 processes)	N/A	14.9	N/A	3.0
Falco	10	8.0	5.9	1.7	1.2
	20	4.7	3.6	1.0	0.8
	30	3.8	2.9	0.8	0.6
	40	3.5	2.6	0.7	0.6

Standalone number of processes indicates the number of FASTQ file pairs that are processed in parallel. Timing for Falco includes initialisation and configuration time which are approximately 10 min. Runtime for STAR with 16 processes is not available as some STAR processes are killed by the operating system, resulting in failure of the job

Runtime comparisons across cluster sizes for alignment with Falco framework shows a decrease in runtime with increasing cluster size (Table 1), indicating the scalability of the alignment-only analysis on the Falco framework. However, the runtime does not linearly decrease with increasing cluster size, with the maximum speedup of 2x achieved by increasing the cluster size from 10 nodes to 20 nodes. The minimal difference in analysis time for cluster ≥20 nodes can partially be attributed to the constant initialisation time and the lack of speed up in the splitting step (Additional file 1), as previously highlighted in the scalability analysis for the initial Falco framework. Another reason for the lack of speedup is due to second stage in the alignment-only step that performs concatenation of the alignment chunks for each sample, meaning that the speedup for this stage is limited by the size of the input files and the subsequent number of read chunks that need to be concatenated. Therefore, the minimal reduction in runtime of the second stage for the mouse and human datasets can be explained by the uneven distribution in the size of the FASTQ files of both the mouse and human datasets, with some samples having input size that is 9x larger compared to the median input size.

### Comparison of Falco alignment-only mode with rail-RNA

As part of the evaluation of the alignment-only analysis using the Falco framework, the performance of Falco was also compared against Rail-RNA, a previously published tool designed for scalable alignment of RNA-seq data developed using the MapReduce programming paradigm. For the comparison, Rail-RNA was configured to output only BAM files in order to reduce the extra processing steps required for producing the default outputs of sample statistics, coverage vectors and junction information. It should be noted that the cluster used for running Rail-RNA utilises a different instance type compared to the cluster used for running Falco (c3.8xlarge for Rail-RNA vs r3.8xlarge for Falco) as Rail-RNA only provides support for a limited number of instance types. To ensure a fair comparison, the instances used for Rail-RNA cluster have the same configuration for CPU, storage and network performance as the instance used for Falco cluster, with the only difference being the memory configuration.

Rail-RNA was able to perform alignment of the human brain dataset in about 6 h using a 40 node cluster, increasing to 16 h using a 10 node cluster. In contrast, Falco was able to perform alignment of the human brain dataset in less than 1 h using a 40 node cluster and in about 2 h using a 10 node cluster, representing a speed up of around 10x compared to Rail-RNA (Table 2). The type of alignment file produced by Rail-RNA differs from that produced by the Falco framework as Rail-RNA by default produces a single alignment file for each chromosome per sample, meaning that users will have to manually combine the alignment files in order to get a single alignment file per sample. While Rail-RNA does provide an option to produce a single alignment file per sample, toggling this option resulted in Rail-RNA failing to complete during BAM writing step. The use of the MapReduce paradigm also means that Rail-RNA produces a lot more intermediate files compared to the Falco framework, with Rail-RNA producing 2.4 TB of intermediate files for alignment of the 220 GB human brain dataset. In comparison, Falco framework only produced a maximum of 200 GB of intermediate files (alignment chunks) for the alignment of the same dataset.

**Table 2:** Runtime comparison for alignment of the human brain single cell dataset using Rail-RNA and Falco frameworks

Nodes	Rail-RNA	Falco	
		STAR	HISAT
10	15.9	5.9	1.2
40	5.7	2.6	0.6

### Evaluation and application of Falco transcript assembly mode

As with the alignment-only mode, the output produced by Falco alignment-only mode was first checked to see if it matches the output produced from single-node analysis. For this test, three different pipeline configurations were evaluated – STAR + StringTie with reference, STAR + Scallop and HISAT + StringTie without reference – using both simulated data and samples from human and mouse single-cell RNA-seq datasets. The simulated data is used to evaluate the performance of the pipelines tested in recovering transcripts from reference annotations, while the 100 randomly selected human and mouse single-cell RNA-seq datasets are used to evaluate the concordance between the assembled transcripts. Concordance evaluation between the output produced by Falco and single-node analysis is performed by comparing the accuracy of the assembled transcript against the reference annotation as reported by the GffCompare tool. GffCompare measures accuracy of the assembled transcripts using two metrics - sensitivity, which is defined as the ratio between the number of correctly assembled transcripts and the total number of transcripts in the reference annotation; and precision, which is defined as the ratio between the number of correctly assembled transcripts and the total number of assembled transcripts. A transcript is determined by GffCompare as correct if there is an 80% overlap for a single-exon transcript or if there is a transcript with a matching intron chain sequence in the reference annotation for a multi-exon transcript.

For the simulated dataset, Polyester [28] was used to generate a 100-bp paired-end human synthetic RNA-seq dataset, with 1000 reads samples for each gene with zero-error rate. In order to evaluate the ability of the pipelines to recover transcripts from the reference annotation, assembled transcripts prior to merging with reference annotation were used for comparison to the reference annotation with GffCompare. From the statistics of the transcript assembled from single node run (Table 3), it can be seen that reference-guided transcript assembly (STAR + StringTie with reference) has a high sensitivity and precision across all features. This is unlike the de novo transcript assembly approaches (STAR + Scallop and HISAT + StringTie) which have high sensitivity and precision for base, exon, intron and locus, but very low precision on intron chain and transcript level. The low accuracy rate of intron chain and transcript features for the de novo approaches can be explained by the limitations of the Polyester tool, which is unable to generate reads with the correct intron chain when using the reference annotation GTF file as input.

**Table 3:** Accuracy of assembled transcripts for simulated data from single node runs

Feature	STAR + StringTie (with reference)		STAR + Scallop		HISAT + StringTie	
	Sensitivity (%)	Precision (%)	Sensitivity (%)	Precision (%)	Sensitivity (%)	Precision (%)
Base	97.3	99.8	87.7	85.2	80.0	93.8
Exon	97.3	98.4	57.0	75.2	63.9	89.9
Intron	96.8	99.3	70.7	99.1	85.7	97.8
Intron Chain	93.6	84	31.9	56.7	25.7	38.9
Transcript	94.1	85.7	33.9	35.2	28.4	43.8
Locus	98.3	99.4	71.9	57.7	69.1	82.0

Comparison of the statistics for transcripts produced by the Falco transcript assembly mode (Table 4) against single-node runs shows differences between the result of the transcript assembly processes, though the results do share a high degree of concordance. For the reference-guided transcript assembly pipeline, the transcripts assembled by the Falco framework have lower sensitivity and precision compared to the single node runs due to the higher number of missed features. In contrast, the transcripts assembled using de novo transcript assembly pipelines on Falco have a slightly higher sensitivity and precision for exon, intron and locus features, as there are less features missed and less novel features introduced. However, the result of de novo transcript assembly approaches also have a lower sensitivity and precision for intron chain and transcript features due to the presence of more assembled transcripts. The difference between the statistics for transcripts assembled using Falco and single-node runs can likely be attributed to the binning approaches utilised by the transcript assembly step in Falco, which may result in partially assembled transcripts in cases where the transcripts spans multiple bins. As seen from the result of transcript assembly with Falco, this issue is more prevalent in the de novo transcript assembly approaches as there is no reference annotation present to repress the creation of partial transcripts.

**Table 4:** Accuracy of assembled transcripts for simulated data from Falco-based runs

Feature	STAR + StringTie (with reference)		STAR + Scallop		HISAT + StringTie	
	Sensitivity (%)	Precision (%)	Sensitivity (%)	Precision (%)	Sensitivity (%)	Precision (%)
Base	96.2	99.9	88.6	85.3	81.1	93.8
Exon	96.4	97.9	59.6	74.7	65.8	86.0
Intron	95.5	99.3	74.5	99.2	87.6	97.8
Intron Chain	92.8	83.1	33.4	50.4	26.7	33.4
Transcript	93.3	84.9	35.2	33.4	29.3	38.0
Locus	98.1	99.3	72.5	59.7	69.7	82.4

To evaluate the performance of the transcript assembly mode on real scRNA-seq datasets, 100 samples were again randomly selected from each of the human brain and mouse embryonic stem cell datasets, as per the test performed during evaluation of the alignment-only mode. Since the datasets are composed of multiple samples, we compared the performance of Falco’s transcript assembly mode against two alternative assembly strategies using: transcript assembly based on Falco-aligned reads from individual samples, followed by merging of all assembled transcripts (individual approach); and perform transcript assembly on a pool of all Falco-aligned reads from all samples (pooled approach). While previous studies have shown that the pooled transcript assembly approach will result in less discovery of novel transcripts from the samples [29], the issues associated with the nature of scRNA-seq necessitate the need to pool reads across samples in order to ensure full recovery of transcript. However, the individual transcripts assembly approach may still be applicable for scRNA-seq data as the assembled transcripts from each sample will be merged together using StringTie merge mode, which is able to collapse transcripts within the same region into a representative transcript across the entire dataset. In both the individual and pooled transcript assembly approaches, we utilised the Falco alignment-only mode to accelerate alignment of the 100 samples, prior to running transcript assembly manually. For this evaluation, GffCompare is run on transcript assembly after merging with the reference annotation, meaning that the sensitivity of the assembled transcripts will be identical across all three transcript assembly approaches (individual, pooled, and Falco-assembly) as the reference transcripts will necessarily be present within the assembled transcript. As such, only the precision metric is usable for accuracy comparison as this metric is calculated based on the total number of transcripts assembled, which will differ across the different approaches.

Comparison of the precision metrics of the individual and pooled transcript assembly approaches shows differences in accuracy depending on the tool used for transcript assembly. StringTie is shown to perform better with the pooled approach due to the lower number of transcripts assembled with the pooled approach, which is consistent with previous work. In contrast, Scallop performs better with the individual transcript assembly approach as it assembled more transcripts in the pooled iterative approach (Tables 5 and 6). This difference in the performance of the two transcript assembly tools can be attributed to the different transcript algorithms used by the tools and the different threshold used for transcript reconstruction. When comparing the individual and pooled approaches against Falco transcript assembly mode, it can be seen that the performance of Falco transcript assembly mode falls in between the individual and pooled approaches, with StringTie based pipelines having more similarity to the individual approach and the Scallop based pipeline having more similarity to the pooled approach (Tables 5 and (6)). These results indicate that the transcripts assembled using Falco transcript assembly mode are similar to those produced with the pooled approach from which the transcript assembly mode is modelled after, though the binning approach utilised for parallelisation of the transcript assembly process does result in more transcripts being assembled. In particular, the bins are configured by default to overlap each other to ensure full assembly of transcripts which are located near the start or end of the bin in at least one of the bins (see Additional file 2). While this does result in redundant copies of transcripts in regions where bins overlap, these redundant transcripts were designed to be removed during the merging step with the reference annotation. However, StringTie merge does not seem to correctly remove these redundant transcripts and instead treat the redundant transcripts as novel isoforms, thereby artificially increasing the number of novel transcripts assembled. Nonetheless, it is not difficult to apply a post-processing step to remove such redundant transcripts. This should improve the precision of the call.

**Table 5:** Precision of assembled transcripts for human brain single cell dataset across different transcript assembly approaches

Feature	STAR + StringTie (with reference)			STAR + Scallop			HISAT + StringTie		
	Falco alignment + individual assembly (%)	Falco alignment + pooled assembly (%)	Falco transcript assembly mode (%)	Falco alignment + individual assembly (%)	Falco alignment + pooled assembly (%)	Falco transcript assembly mode (%)	Falco alignment + individual assembly (%)	Falco alignment + pooled assembly (%)	Falco transcript assembly mode (%)
Base	42.7	63.0	41.8	48.9	27.9	28.9	40.6	60.1	38.9
Exon	76.5	92.3	79.0	79.4	73.1	73.4	72.9	90.2	76.0
Intron	88.0	96.9	92.3	88.7	91.7	91.1	82.9	94.6	88.2
Intron Chain	79.1	94.5	85.9	80.1	84.4	83.3	72.1	90.7	79.6
Transcript	57.5	83.0	60.2	62.1	51.0	51.6	54.8	80.9	57.8
Locus	32.2	61.5	32.4	37.2	24.1	24.9	30.5	58.9	31.2

**Table 6:** Precision of assembled transcripts for mouse embryonic stem cell single cell dataset across different transcript assembly approaches

Feature	STAR + StringTie (with reference)			STAR + Scallop			HISAT + StringTie		
	Falco alignment + individual assembly (%)	Falco alignment + pooled assembly (%)	Falco transcript assembly mode (%)	Falco alignment + individual assembly (%)	Falco alignment + pooled assembly (%)	Falco transcript assembly mode (%)	Falco alignment + individual assembly (%)	Falco alignment + pooled assembly (%)	Falco transcript assembly mode (%)
Base	52.8	90.3	55.0	56.2	34.5	38.4	51.1	88.0	51.0
Exon	69.8	95.9	78.3	74.6	71.7	73.1	69.1	95.3	76.2
Intron	77.0	96.9	88.0	80.9	87.1	85.6	76.0	96.4	86.3
Intron Chain	57.0	93.4	76.1	62.9	73.0	70.5	56.4	92.3	73.1
Transcript	48.1	90.9	58.8	54.8	47.8	50.8	48.5	90.2	56.2
Locus	40.4	86.1	41.7	46.3	30.1	33.9	40.1	85.8	39.5

### Scalability of Falco transcript assembly mode

The performance evaluation of the transcript assembly mode is done in a similar manner to the performance evaluation of alignment-only mode, with runtime comparison of transcript assembly on a single node with varying degrees of parallelisation against the runtime of transcript assembly on the Falco framework with differing cluster sizes. For the single node runs, the individual transcript assembly approach was used for performing transcript assembly of the scRNA-seq datasets, with both the alignment and transcript assembly of individual samples being run in a single node. The runtime of the single node runs are calculated based only on the timings for the alignment and transcript assembly process, while the runtime for the Falco transcript assembly process includes both cluster initialisation time and FASTQ splitting steps like before.

The runtime of transcript assembly for the human brain dataset on a single node ranges from 4.1 h (HISAT + StringTie, 16 processes) up to 17.2 h (STAR + StringTie with reference, 1 process). In comparison, the time taken for transcript assembly of the human dataset using the Falco framework is at most 2.9 h (STAR + StringTie with reference, 10 nodes), with a minimum time of 0.9 h (HISAT + StringTie, 40 nodes) (Table 7). Runtime comparisons between the same pipeline across the single node and Falco runs shows that Falco is able to achieve an average speed up ranging from 1.7x (12/16 processes vs 10 nodes) up to 16.5x (1 process vs 40 nodes). Furthermore, runtime comparison for Falco across increasing cluster size also shows the scalability of the transcript assembly mode in Falco, with a maximum speedup of 1.7x when increasing the cluster size from 10 nodes to 20 nodes (Table 7). The reduction in speedup for clusters with >20 nodes is again due to a number of factors, including the constant cluster start up time and lack of speed up in the FASTQ splitting step due to the uneven distribution of file sizes (Additional file 3). There is also a limiting factor specific to the transcript assembly step as a result of an uneven distribution of bin sizes, with the largest bin in the human dataset having close to 20 million reads (Additional file 4).

**Table 7:** Runtime comparison for transcript assembly of single cell dataset with and without the Falco framework

System	Nodes	Human - brain (hours)		
		STAR + StringTie (with reference)	STAR + Scallop	HISAT + StringTie
Standalone	1 (1 process)	17.2	16.2	16.3
	1 (12 processes)	4.2	5.5	5.7
	1 (16 processes)	N/A	N/A	4.1
Falco	10 node	2.9	2.8	2.3
	20 node	1.7	1.7	1.4
	30 node	1.3	1.3	1.1
	40 node	1.1	1.1	0.9

Standalone number of processes indicates the number of FASTQ file pairs that are processed in parallel. Timing for Falco includes initialisation and configuration time which are approximately 10 min. Runtime for STAR with 16 processes is not available as some STAR processes are killed by the operating system, resulting in failure of the job

The performance analysis of Falco transcript assembly mode does not include the timings for the mouse ESC dataset as this dataset fails to complete when run using the Falco framework. There are two different types of issues that results in the failure of the transcript assembly step - memory (RAM) and serialisation. The memory issue was encountered during execution of transcript assembly using Scallop on the third largest bin in the mouse dataset, which contains around 11 million reads (Additional file 4). Due to the large number of alignment records that need to be processed, Scallop uses up more memory beyond the assigned 30 GB of memory, resulting in the executor being killed as there is a strict limit imposed by the YARN resource manager on the memory consumption of executors. The serialisation issue, on the other hand, was encountered when performing transcript assembly using StringTie on the two largest bins in the mouse dataset, containing around 30 million reads each (Additional file 4), and is a result of the limitation of the PySpark framework used for developing the Falco framework. In both PySpark and Spark frameworks, the input and output from executors undergo a serialisation process to allow for data to be transferred to different executors and nodes. For the PySpark framework, the serialisation process is handled by the built-in Pickle library and there is a limitation in the size of data that can be serialised. The size of the data for the two largest bins exceeds the maximum limit of the data that can be serialised by the Pickle library, therefore resulting in an error during reading of the alignment records into the executor.

The two issues encountered during processing of the mouse dataset are primarily a result of the large input size of the dataset and the need to pool together reads for performing transcript assembly. One approach that can be used to process such a large dataset is to divide the samples into smaller batches and perform transcript assembly on these smaller batches, followed by merging of the assembled transcripts from each batch together to obtain a consensus set of transcripts across the full dataset. This approach will require some manual intervention from the user as they need to perform batching and merging of the assembled transcripts. The batching process may also result in some transcripts being missed as there will only be partial information available during the transcript assembly process. Another possible approach, which is applicable for memory-related failures, is to increase the amount of memory assigned to the executors beyond the default 30 GB limit, though this will result in a reduced number of available executors. A third approach is to utilise Falco alignment-only mode to perform read alignment of the samples, followed by transcript assembly on the merged read alignments. This last approach will also require some manual intervention from the user as they need to merge the alignment results and perform transcript assembly.

## Conclusion

The Falco framework extends the capability of the existing Falco framework through the addition of two processing modes - alignment-only and transcript assembly modes. The alignment-only mode is designed to enable other types of RNA-seq analysis beyond gene expression analysis by producing read alignment output (in BAM format), which can then be used as input for further downstream analysis tools. As with the initial framework, the alignment-only mode on Falco is configurable by the user and currently supports STAR and HISAT2 as the alignment tool. From benchmarks using two single-cell RNA-seq datasets, we show that Falco is able to speed up the alignment process by 2.5 to 16.4 times compared to alignment using a single node with highly-optimised execution while still producing identical alignment output. Comparison of Falco alignment only mode against a similarly designed tool, Rail-RNA, shows the capability of Falco in performing alignment in a time-efficient manner, with Falco achieving an average of 10 times speed up compared to Rail-RNA. There are, however, some limitations with the alignment-only mode in terms of the scalability and storage due to limitations with the Spark framework used and the need to produce a single alignment file per sample so users will not need to merge the result of the alignment manually.

The addition of transcript assembly mode in the Falco framework is designed to allow for the identification of novel transcripts by creating a more accurate gene annotation from the scRNA-seq dataset being analysed. Unlike in the alignment-only mode of Falco and the gene expression analysis mode of Falco frameworks, the transcript assembly mode requires a different approach for parallelising transcript assembly as this process requires reads from the same region to be processed together. Using both simulated and real scRNA-seq datasets, we show that the binning-based approach used by Falco transcript assembly mode is able to successfully perform transcript assembly, with a high concordance between the transcripts assembled with Falco and transcripts assembled using two iterative transcript assembly approaches. Of the two single-cell RNA-seq datasets tested, Falco transcript assembly was able to speed up the analysis of the human brain dataset by a minimum of 1.7 times, up to a maximum of 16.5 times. Unfortunately, Falco transcript assembly mode failed to perform transcript assembly for the larger mouse ESC dataset due to a number of factors, including high memory usage of the Scallop transcript assembly tool and a limitation with the amount of data that can be handled by the PySpark framework. There are a few approaches which can be used for processing such large datasets, including dividing and processing the data in smaller batches, providing more memory to the worker, and using Falco alignment-only mode for alignment, prior to assembly. Regardless, we show that the Falco transcript assembly mode is able to perform transcript assembly in a scalable manner and is also able to identify novel transcripts present within scRNA-seq datasets.

## Availability and requirements

**Project name**: Falco

**Project home page**: https://github.com/VCCRI/Falco

**Operating system(s)**: Linux, Mac OS X (command-line software)

**Programming language**: Python

**Other requirements**: Apache Spark

**License**: GPL-3

**Any restrictions to use by non-academics**: none

## Supplementary information

**Additional file 1** Supplementary Figure 1. Falco alignment-only processing time split by steps and stages for STAR and HISAT2 pipelines in the analysis of mouse embryonic stem cell and human brain single cell data. The timings shown do not include cluster initialisation time, as it is constant across differing cluster sizes.

**Additional file 2** Supplementary Material. Considerations for the implementation of Falco alignment-only and transcript assembly modes.

**Additional file 3** Supplementary Figure 2. Falco transcript assembly processing time split by steps and stages for STAR + StringTie with reference, STAR + Scallop, and HISAT2 + StringTie pipelines in the analysis of human brain single cell data. The timings shown do not include cluster initialisation time, as it is constant across differing cluster sizes, or later stages of the transcript assembly step, as the total time taken for the remaining steps is <30 s.

**Additional file 4** Supplementary Figure 3. Distribution of read bin sizes for (A) human brain and (B) mouse embryonic stem cell single cell datasets.

## Data Availability

All data underlying the results are available as part of the article and no additional source data are required.

## Abbreviations

EMR: Elastic MapReduce
GTF: General transfer format
HDFS: Hadoop distributed file system
mRNA: Messenger RNA
RNA-seq: RNA sequencing
RNA: Ribonucleic acid
S3: Simple storage service
scRNA-seq: Single-cell RNA sequencing
