# Supplementary figures and images for: Cloud accelerated alignment and assembly of full-length single-cell RNA-seq data using Falco
Source: BMC Genomics. 2019 Dec 30;20(Suppl 10):927. doi: 10.1186/s12864-019-6341-6 (PMC6936136; doi:10.1186/s12864-019-6341-6)

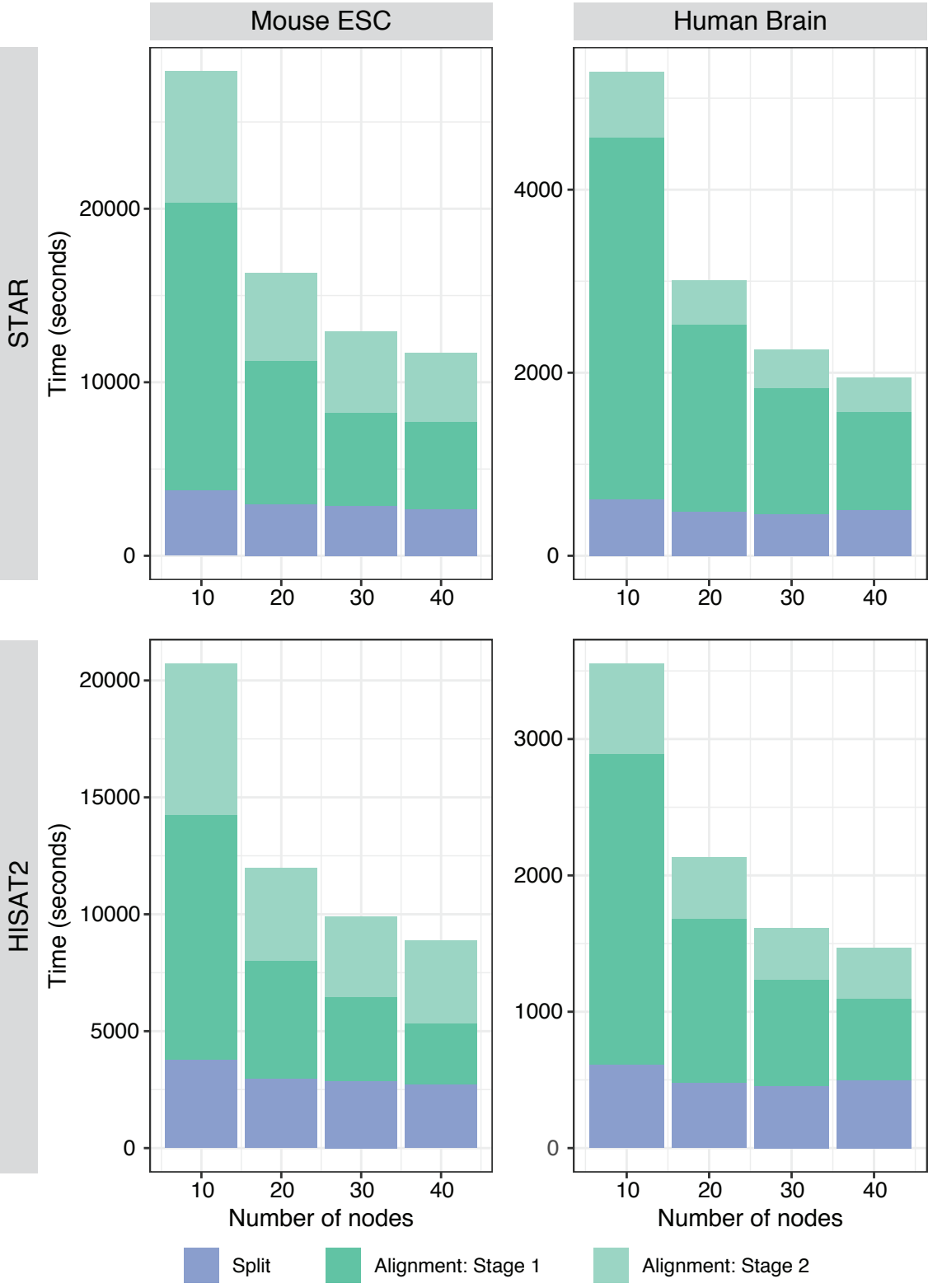

Supplement: Supplementary file 1 — Additional file 1 Supplementary Figure 1. Falco alignment-only processing time split by steps and stages for STAR and HISAT2 pipelines in the analysis of mouse embryonic stem cell and human brain single cell data. The timings shown do not include cluster initialisation time, as it is constant across differing cluster sizes. [file 12864_2019_6341_MOESM1_ESM.pdf]

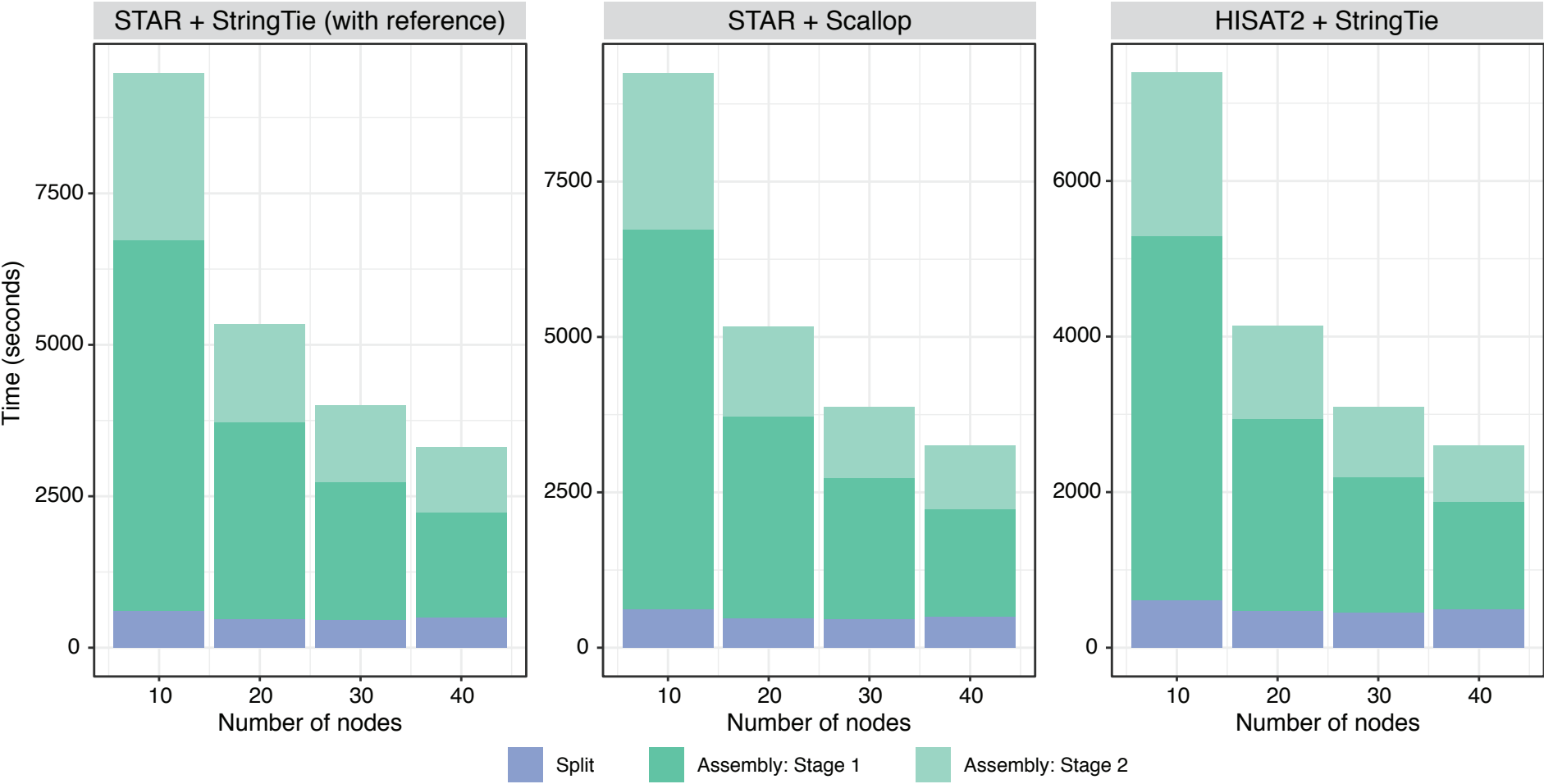

Supplement: Supplementary file 3 — Additional file 3 Supplementary Figure 2. Falco transcript assembly processing time split by steps and stages for STAR + StringTie with reference, STAR + Scallop, and HISAT2 + StringTie pipelines in the analysis of human brain single cell data. The timings shown do not include cluster initialisation time, as it is constant across differing cluster sizes, or later stages of the transcript assembly step, as the total time taken for the remaining steps is <30 s. [file 12864_2019_6341_MOESM3_ESM.pdf]

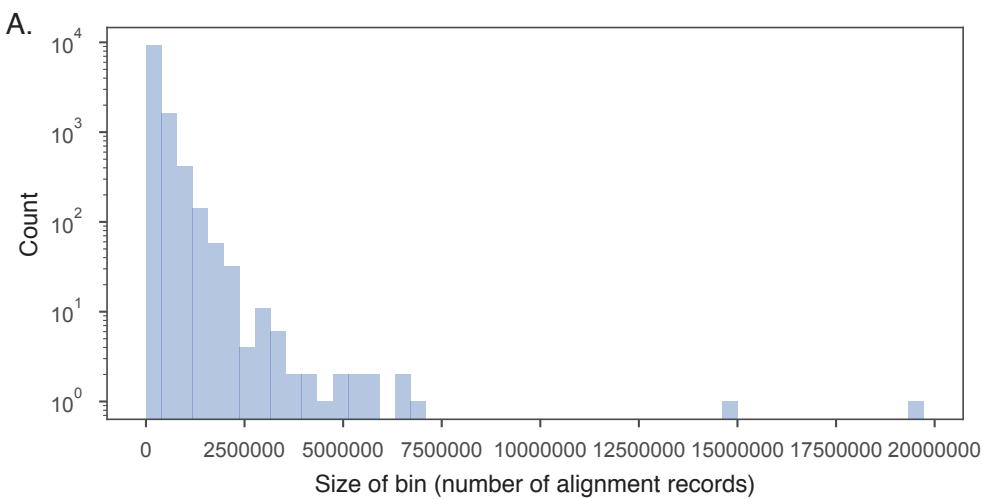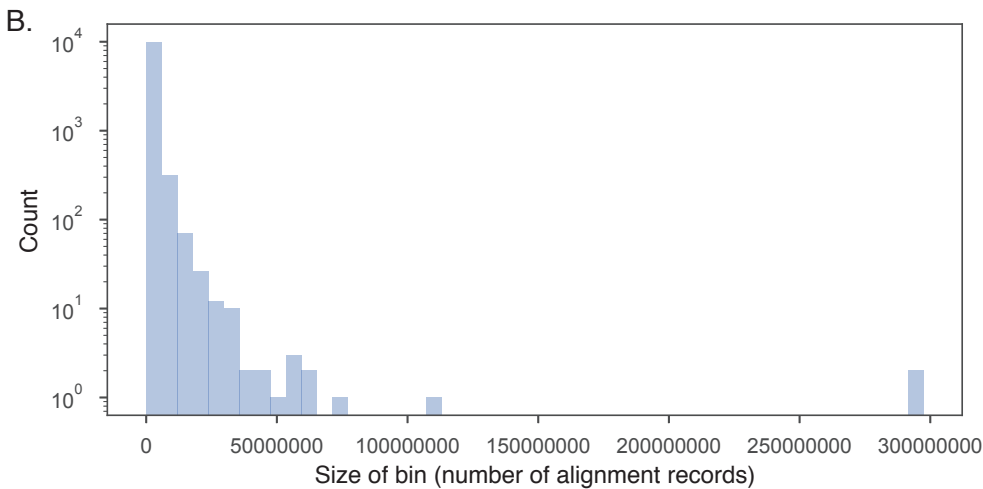

Supplement: Supplementary file 4 — Additional file 4 Supplementary Figure 3. Distribution of read bin sizes for (A) human brain and (B) mouse embryonic stem cell single cell datasets. [file 12864_2019_6341_MOESM4_ESM.pdf]
