# Supplementary material for: Cloud accelerated alignment and assembly of full-length single-cell RNA-seq data using Falco
Source: BMC Genomics. 2019 Dec 30;20(Suppl 10):927. doi: 10.1186/s12864-019-6341-6 (PMC6936136; doi:10.1186/s12864-019-6341-6)
Supplement: Supplementary file 2 — Additional file 2 Supplementary Material. Considerations for the implementation of Falco alignment-only and transcript assembly modes. [file 12864_2019_6341_MOESM2_ESM.pdf]

SUPPLEMENTARY MATERIALS

Cloud accelerated alignment and assembly of full-length single-cell RNA-seq data using Falco

Andrian Yang[1,2], Abhinav Kishore[1], Benjamin Phipps[1] and Joshua WK Ho[1,2,3*]

Consideration for alignment-only mode

The current approach used in the alignment step is not the most efficient approach, particularly due to the writing and reading cost incurred from storing the alignment chunks in a temporary location on HDFS or S3 in between the two stages of the alignment step. One of the more efficient approaches which was explored during early development of the alignment step is to output the alignment records during the alignment stage, followed by the shuffling process to combine all alignment records for each sample, before writing the combined aligned records for each file into a single alignment file and uploading it to the output location. However, this approach suffers from memory issues due to the requirement of producing a single alignment file per sample, as a sample can have an arbitrary amount of read chunks, and the strict memory management of the Spark executor by the YARN resource manager, which will kill the executor if it utilises more memory than is allocated.

One possible way to alleviate the memory issue is to produce multiple alignment files per sample (i.e. grouped by chunks or by chromosome), though this approach will require users to manually merge the alignment files by themselves which is not ideal, especially for large number of samples. Another potential solution which was explored is to increase the amount of memory allocated to the executor so that the executor can handle a larger amount of input. This approach unfortunately did not alleviate the issue of the executor being killed and is also not practical as the memory issue may occur again with even larger file sizes. Furthermore, by increasing the memory allocated per executor, it will reduce the amount of executors available therefore reducing the processing capability of the cluster and increasing the analysis time. Given the limitations of the more efficient approach, we elected to use the current approach of storing alignment chunks in a temporary location in HDFS or S3 as this approach will not result in memory issues due to the small amount of output produced (i.e. location of alignment chunks rather than entire alignment records) and does not require the input FASTQ file to have a certain file size limit.

*Correspondence: jwkho@hku.hk
[1]Victor Chang Cardiac Research Institute, 405 Liverpool St, 2010, Darlinghurst, Australia
Full list of author information is available at the end of the article

Consideration for transcript assembly mode

Transcript assembly requires a different parallelisation approach compared to the alignment and read quantification analysis. In transcript assembly, reads from the same region – such as genes – will need to be processed together to allow for sharing of information from different reads in order to construct the transcript itself. This is in contrast to the alignment and read quantification analysis where reads can be processed independently of each other during both the alignment and quantification process. Given this constraint, the aligned reads will need to first be grouped together prior to the transcript assembly process. Here, we consider a number of approaches that can be utilised to parallelise transcript assembly of multiple samples while still allowing for sharing of information between reads.

A simple and naive approach for parallelising transcript assembly is to first group the reads by sample, followed by performing transcript assembly across samples in parallel. This approach can easily be implemented by extending the previous alignment step to perform transcript assembly after concatenation of alignment chunks. However, this approach has limitations in terms of the parallelisation capability as the amount of parallelisation that can be achieved during the transcript assembly processing is dependent on the amount of samples in the dataset. This approach is also not suitable for the type of data to be analysed - primarily scRNA-seq datasets - due to the low amount of reads sequenced per cell (sample), which may limit the ability of the transcript assembly tool to assemble transcripts as there may not be enough reads to support the transcript, and the possibility of drop-out, where lowly expressed genes/transcripts are not captured and sequenced in a cell. In order to alleviate the issues inherent to scRNA-seq datasets, we will need to perform transcript assembly using the combined reads from all samples.

The need to combine reads across samples means that the approaches for parallelising transcript assembly will need to be based on segmenting the genome into sections which can be processed in parallel. A natural approach to divide the genome is to use chromosomes for segmentation. This approach is particularly advantageous as transcript assembly between different chromosomes can be done independently of each other.

However, there are typically only a small number of chromosomes in the genome of an organism, particularly in genomes of reference organisms, therefore the amount of parallelisation that can be achieved is somewhat limited. The approach used in the implementation of the transcript assembly step is to divide the genome into smaller regions – or bins – to maximise the parallelisation of the transcript assembly process. Ideally, the bins should be created based on regions where reads are clustered, which indicates the presence of transcripts. Nonetheless, performing clustering of reads to create bins is a computationally expensive process and therefore the binning strategy used in the transcript analysis step is to create identically sized bins across the entire genome. This binning strategy does have a disadvantage of potentially separating transcripts across bins and thus producing partial non-overlapping transcripts. To solve this issue, the bins are made to overlap each other by at least the length of the longest gene in human – Titin – to ensure that at least one bin can produce the full length transcript. Moreover, a merging step is performed after transcript assembly of all bins with the reference annotation using StringTie in order to remove redundant transcripts and to produce an updated annotation file containing both reference transcript and novel transcript.

Author details

[1]Victor Chang Cardiac Research Institute, 405 Liverpool St, 2010, Darlinghurst, Australia. [2]St Vincent's Clinical School, University of New South Wales, , 2010, Darlinghurst, Australia. [3]School of Biomedical Sciences, Li Ka Shing Faculty of Medicine, The University of Hong Kong, Hong Kong, China.

References
